# Characterisation of UK diets according to degree of food processing and associations with socio-demographics and obesity: cross-sectional analysis of UK National Diet and Nutrition Survey (2008–12)

**DOI:** 10.1186/s12966-015-0317-y

**Published:** 2015-12-18

**Authors:** Jean Adams, Martin White

**Affiliations:** Centre for Diet & Activity Research, MRC Epidemiology Unit, Institute of Metabolic Science, Cambridge Biomedical Campus, University of Cambridge School of Clinical Medicine, Box 285, Cambridge, CB2 0QQ UK

**Keywords:** Body weight, Food supply, Obesity, Socioeconomic factors

## Abstract

**Background:**

Food processing alters food from its natural state for safety, convenience, taste or palatability. Previous research suggests that industrially processed foods, and diets high in these products, tend to be less healthful. However, most previous work is based on household, rather than individual-level, data. Little has been reported on the relationship between processed food consumption and markers of health; or on socio-demographic correlates of processed food consumption.

Our objective was to describe: the nutritional content of foods classified according to degree of processing; the nutritional content of diets with different relative intakes of processed foods; the socio-demographic characteristics of individuals with different relative intakes of processed foods; and the association between intake of processed foods and body weight.

**Methods:**

Secondary analysis of data from the UK National Diet and Nutrition Survey (2008–12), a large national cross-sectional study of diet. Dietary information was collected using four-day, unweighed, food-diaries. Foods were classified as: unprocessed or minimally processed (MPF; foods with no processing or mostly physical processes applied to single whole foods), processed ingredients (PI; extracted and purified components of single whole foods), or ultra-processed food products (UPF; products produced from industrial combining of MPF and PI).

**Results:**

Two thousand one hundred seventy four adults were included. MPF and diets high in these foods, had the most healthful nutritional profile. UPF did not necessarily have the least healthful nutritional profile, but diets high in these foods did. Women, and older adults consumed more energy from MPF, and less from UPF. Those living in lower occupation social class households consumed less energy from MPF, but no more from UPF. Only higher intake of PI was consistently, inversely, associated with body weight.

**Conclusions:**

This is the first study to explore correlates of processed food consumption, using individual-level data from a large, national sample. Although higher intakes of MPF and lower intakes of UPF were associated with the most healthful dietary profiles, only intake of PI was consistently associated with body weight. Consumption of UPF varied by age and gender, but, unexpectedly, not by occupational social class. Longitudinal work is required to confirm relationships with health markers.

**Electronic supplementary material:**

The online version of this article (doi:10.1186/s12966-015-0317-y) contains supplementary material, which is available to authorized users.

## Background

Food processing alters food from its natural state for safety, convenience, taste or palatability and has been identified as both a key contributor to human development [1, 2] and a substantial threat to health [3, 4]. As food processing includes industrial processes and home preservation [5], processed food varies widely. A number of typologies have been developed to categorise products according to their degree of processing [6]. We focus here on industrially processed foods [7] (and refer to these as ‘processed foods’ herewith) as they are likely to make a larger contribution to diets in developed countries than domestically processed foods.

One commonly used typology for classifying foods according to degree of industrial processing, that has been suggested as particularly specific, coherent, clear, comprehensive and workable [6], is described in Table 1 [7]. This describes three groups of foods. Unprocessed, or minimally processed, foods (MPF) are single whole foods that have not been processed or been subject to simple physical processes to make them more durable, accessible, convenient, palatable or safe. Processed ingredients (PI) are extracted or purified components of single whole foods that are generally used in the preparation of dishes, or the production of ultra-processed food products (UPF). Ultra-processed food products are made by processing together MPF with PI.

**Table 1:** Food classification based on the extent and purpose of industrial processing; from [7]

Group	Definition	Examples
Group 1: Unprocessed or minimally processed foods	No processing or mostly physical processes used to make single whole foods more durable, accessible, convenient, palatable or safe.	Fresh, chilled, frozen, vacuum-packed fruits, vegetables, fungi, roots and tubers; grains (cereals) in general; fresh, frozen and dried beans and other pulses (legumes); dried fruits and 100 % unsweetened fruit juices; unsalted nuts and seeds; fresh, dried, chilled, frozen meats, poultry and fish; fresh and pasteurized milk, fermented milk such as plain yoghurt; eggs; teas, coffee, herb infusions, tap water, bottled spring water.
Group 2: Processed ingredients	Extraction and purification of components of single whole foods, resulting in producing ingredients used in the preparation and cooking of dishes and meals made up from Group 1 foods in homes or traditional restaurants, or else in the formulation by manufacturers of Group 3 foods.	Vegetable oils, margarine, butter, milk cream lard; sugar, sweeteners in general; salt; starches, flours, and “raw” pastas and noodles (made from flour with the addition only of water); and food industry ingredients usually not sold to consumers as such, including high fructose corn syrup, lactose, milk and soy proteins, gums, and preservatives and cosmetic additives.
Group 3: Ultra-processed food products	Processing of a mix of Group 2 ingredients and Group 1 foodstuffs in order to create durable, accessible, convenient, and palatable ready-to-eat or to-heat food products liable to be consumed as snacks or desserts or to replace home-prepared dishes.	Breads, biscuits (cookies), cakes and pastries; ice cream; jams (preserves); fruits canned in syrup; chocolates, confectionery (candies), cereal bars, breakfast cereals with added sugar; chips, crisps; sauces; savoury and sweet snack products; cheeses; sugared fruit and milk drinks and sugared and “no-cal” cola, and other soft drinks; frozen pasta and pizza dishes; pre-prepared meat, poultry, fish, vegetable and other “recipe” dishes; processed meat including chicken nuggets, hot dogs, sausages, burgers, fish sticks; canned or dehydrated soups, stews and pot noodle, salted, pickled, smoked or cured meat and fish; vegetables bottled or canned in brine, fish canned in oil; infant formulas, follow-on milks, baby food.

Ultra-processed food products, and diets high in these, tend to be more energy dense and higher in fat, saturated fat, salt and sugar, and lower in fibre than is optimal for health [5, 7, 8]. For this reason, diets high in UPF have been described as “intrinsically nutritionally unbalanced and intrinsically harmful to health” (p730) [9]. However, few studies provide strong evidence that such foods are harmful to health. Ultra-processed food products can make significant contributions to nutrient intake [5, 8, 10–12] and wide variations in the nutritional content of processed foods have been reported [5]. Few authors have explored direct associations between UPF consumption and health markers. However, one study found body mass index (BMI) was associated with higher intake of UPF in Brazil [13]. Another found greater consumption of UPF in Brazilian adolescents with metabolic syndrome compared to those without [14].

The majority of recent studies on the contribution of UPF to overall diets have made use of household budget surveys [5, 7, 8, 11–13, 15–17]. These tend to exclude food purchased and consumed out-of-home meaning they do not capture the totality of diet. Household budget surveys also provide information at the household, rather than individual, level making the assumption that all members of a household consume similar diets. This can make it difficult to draw conclusions about associations with health and disease outcomes. Nevertheless, according to these surveys, the percentage of energy derived from UPF appears to have increased over time and be greater in more, compared to less, developed countries [11, 12, 15, 16]. Whilst consumption of UPF was positively associated with income in Brazil [7, 15], it was not in Canada [8] – possibly due to the greater relative affordability of these foods which has been observed in more developed countries [11], contributing to ‘nutrition transition’ [18]. Little else is known about how consumption of processed foods varies within populations.

We used population-based individual level data from the UK to fill the gaps in existing knowledge identified above and overcome limitations of previous work using household budget surveys. Specifically, the aims were to describe: the nutritional content of UK foods classified according to degree of processing; the nutritional content of UK diets with different relative intakes of processed foods; the socio-demographic characteristics of UK individuals with different relative intakes of processed foods; and the association between intake of processed foods and overweight and obesity in the UK.

### Subjects and methods

We conducted a secondary analysis of cross-sectional data from the UK National Diet and Nutrition Survey (NDNS), 2008-12.

### Data source

We used data from waves 1–4 of the NDNS. The NDNS is an annual, cross sectional survey collecting information on the nutrient intakes and status of individuals living in private households in the UK. The current ‘rolling programme’ began in 2008-09 and recruits around 500 adults per year. Methods stay constant from year to year allowing data to be combined across years.

Households across the UK are selected to take part in NDNS using multi-stage probability design. In each year, a random sample of ‘primary sampling units’ are selected for inclusion. These are small geographical areas that allow more efficient data collection by enabling it to be geographically focused. Within these primary sampling units, private addresses are randomly selected from the Postcode Address File (a list of all addresses in the UK) for inclusion. If, on visiting, it is found that more than one household lives at a particular address, one is randomly selected for inclusion. Within participating households, up to one adult and one child are randomly selected to take part. Data collection involves a researcher interview covering socio-demographics and shopping, cooking and eating habits; participant completion of a four-day food diary (with days selected to ensure even representation of all days of the week across the whole sample); and a nurse visit [19].

Recruitment procedures involved sending a letter and leaflet describing the study to selected households a few days before an interviewer visited the household in person. All participants who completed at least three of the four food diary days were thanked with £30 (approx. E41.20, US$46.60) of high street shopping vouchers.

Overall, 91% of households eligible for inclusion agreed to take part and individuals for inclusion were selected. Usable food diaries (three or four completed days) were collected from at least one household member in 58% of eligible households. At an individual level, 56% of those selected to take part completed usable food diaries [19]. Study weights (see ‘Data analysis’ below) were used to adjust for sampling and non-response bias.

### Inclusion criteria

Individuals were included in the analyses if they took part in NDNS waves 1–4, were aged 18 years or older at data collection, and completed at least three out of four food diary days.

### Variables of interest

#### Classification of foods based on degree of food processing

We used the three-level categorisation of foods based on the extent and purpose of industrial processing [7] to categorise all foods recorded in NDNS food diaries (see Table 1). More than 5000 individual foods and drinks (referred to as ‘food’ throughout) have been recorded in NDNS food diaries. All foods in NDNS are grouped into main (*n* = 60) and subsidiary food groups (*n* = 153). As far as possible, subsidiary food groups were coded in their entirety according to degree of processing (see Additional file 1). In cases of uncertainty (*n* = 15), all foods within a subsidiary food group were individually coded. An example of where this occurred was the subsidiary food group ‘yoghurt’. As this group contains both unsweetened yoghurt (defined as MPF, see Table 1) and sweetened yoghurt (defined as UPF as it involves the additional of a PI (sugar) to a MPF (unsweetened yoghurt), see Table 1), all foods within the group were individually coded.

Foods in NDNS are not always disaggregated into constituent ingredients. For example, ‘macaroni and cheese’ may be listed, rather than ‘pasta’, ‘cheese’, ‘milk’, and ‘flour’. However, such dishes are identified as homemade or manufactured. As previously [7], homemade dishes were categorised according to the main constituent ingredient brought into the home, as identified in NDNS. Thus ‘macaroni and cheese, homemade’ is listed as a ‘pasta dish’ and so was coded as pasta, and hence a PI. ‘Macaroni and cheese, manufactured’ was coded as UPF.

Coding of food groups and foods according to the degree of food processing was conducted independently by two researchers (JA, MW). Disagreements were resolved by discussion.

#### Nutritional composition of diets

Individual food diary data in NDNS is supplied ready-linked to nutritional data on all foods consumed. This was used to determine the overall composition of diets. Codes indicating degree of food processing were merged into this data to explore differences in diets by relative consumption of foods in the three food processing groups.

#### Socio-demographic variables

Socio-demographic variables were: age in years (grouped into 10 year age-bands for descriptive analyses), gender, and household occupational social class (as a marker of socio-economic position). Household occupational social class was categorised into three groups (routine and manual, intermediate, or managerial and professional occupations) based on the occupation of the highest household earner using the National Statistics Socio-economic Classification [20]. Where no member of the household is currently employed, classification was made based on last main jobs of household members. Many different measures of socio-economic position are available and no perfect measure exists [21, 22]. Other common measures are income and education – both of which are captured in NDNS. We did not use income as questions on this are refused by around 15% of the sample. We did not use education as a marker of socio-economic position as there are marked secular trends in education in the UK (higher educational attainment has become substantially more common over time) [22].

#### Overweight and obesity

Body mass index was calculated from nurse measured height and weight. Those with a BMI of 25 or more were classified as overweight and those with a BMI of 30 or more as obese [23].

### Data analysis

To describe the nutritional content of foods according to their degree of processing, the total weight (in g) of each food in the NDNS database consumed by all NDNS participants combined was calculated. Nutritional content was characterised in terms of: median sodium and fibre density, and percentage of energy from: protein, carbohydrate, fat, saturated fat and sugar.

Associations between socio-demographic characteristics and relative intake of foods in each food processing group (measured as percentage of energy from foods in each group) were explored using multiple linear regression. Here, percentage of total energy derived from foods in each group were the outcome variables and socio-demographic variables (gender, occupational social class, and age) were the simultaneous explanatory variables. Separate models were constructed for each food processing group and all models were additionally adjusted for percentage of energy derived from alcohol.

Multiple linear regression was used to determine the association between relative intake of foods in each food processing group (measured as percentage of energy from foods in each group) and intake of key foods and nutrients: mean daily 80g portions of fruit and vegetables, mean fibre and sodium intake (in g/day and mg/day respectively), and percentage of energy derived from protein, fat, saturated fat, carbohydrate, and free sugars. Here, foods and nutrients were the outcome variables and percentage of energy from food in each food processing groups were the explanatory variables. Separate models were constructed for each food processing group and key food/nutrient combination. All models were adjusted for gender, occupational social class, age and percentage of energy derived from alcohol.

Multiple linear regression was used to determine associations between relative intake of foods in each food processing group (measured as percentage of energy from foods in each group) and BMI. Multiple logistic regression was used to determine whether the odds of overweight and obesity, and obesity, was associated with relative intake of foods from each food processing group. Here measures of body mass were the outcome variables and percentage of energy from food processing groups the explanatory variables. Separate models were constructed for each food processing group and each outcome (BMI, overweight and obesity, obesity) combination. All models were adjusted for gender, occupational social class, age and percentage of energy derived from alcohol.

It has been suggested that PI are rarely eaten alone, but more commonly consumed in combination with MPF [8, 16]. As such, throughout we explored trends and relationships according to each of the three food processing groups (MPF, PI and UPF), as well as according to a fourth group combining MPF and PI. Throughout, study weights, prepared by NDNS and provided with the data, were used to account for any bias introduced by the sampling procedures and selective non-response. These weights take account of the fact that some population groups are less likely to be invited to take part in the survey (particularly those living in households with more than one adult or child) and some population groups are less likely to agree to take part than others. The use of study weights mean that percentages (with 95 % confidence intervals) are presented rather than raw frequencies. All analyses were conducted in Stata v13 on an available case basis.

### Ethics, consent and permissions

All participants provided written informed consent to take part in NDNS. Anonymised data from waves 1–4 (2008–09 to 2011–12) of the NDNS were obtained from the UK Data Archive – a data sharing service for the UK research community. These data are available to other eligible researchers directly from the Archive. Ethical approval for NDNS was obtained from the Oxfordshire A Research Ethics Committee. Additional ethical approval for this secondary analysis of anonymised data was not required.

## Results

In total, 2174 participants met the inclusion criteria and were eligible for inclusion in the analyses. The only missing data was on social class: missing for 87 (4.2 %) participants and on BMI (missing for 183 (8.4 %) participants).

A mean of 28 % of energy was obtained from MPF, 13 % from PI and 53 % from UPF. The socio-demographic and body mass characteristics of participants is described in Table 2. Overall, 64 % were overweight or obese, and 28 % obese.

**Table 2:** Characteristics of sample; UK National Diet & Nutrition Survey, 2008–12

Variable	Level	Distribution, % (95 % CI)^1^
Gender	Male	48.6 (46.2–51.0)
	Female	51.4 (49.0–53.8)
Occupational social class	Managerial & professional	44.1 (41.7–46.5)
	Intermediate	31.0 (28.8–33.3)
	Routine & manual	24.9 (22.9–27.1)
Age group	18–29	19.3 (17.3–21.6)
	30–39	17.0 (15.4–18.8)
	40–49	19.0 (17.3–20.9)
	50–59	15.7 (14.1–17.4)
	60–69	13.8 (12.2–15.4)
	70+	15.2 (13.5–17.0)
Body mass index	Normal weight (BMI <25)	35.9 (33.6–38.4)
	Overweight (BMI 25–29.9)	64.1 (61.6–66.4)
	Obese (BMI >30)	27.9 (25.8–30.2)

^1^ *CI* Confidence intervals

### Nutritional content of foods classified according to degree of processing

The nutritional content of all foods consumed, weighted by relative intake in grams, is shown in Table 3. Unprocessed or minmally processed foods were highest in protein and lowest in energy density, sodium, fat, saturated fat, carbohydrates and free sugars. Processed ingredients were highest in energy density, fat, saturated fat and free sugars and lowest in fibre and protein. Ultra-processed food products were highest in fibre and carbohydrates.

**Table 3:** Median nutritional content of all foods consumed by food-processing group; UK National Diet & Nutrition Survey, 2008–12

	Unprocessed or minimally processed foods, mean (95 % CI)^1^	Processed ingredients, mean (95 % CI)	Unprocessed or minimally processed foods AND processed ingredients, mean (95 % CI)	Ultra-processed, mean (95 % CI)	All foods, mean (95 % CI)
Energy, kJ/100g	211 (209–213)	1871 (1859–1883)	528 (523–532)	1003 (997–1008)	666 (663–669)
Fibre, g/100g	0.6 (0.6–0.6)	0.4 (0.3–0.4)	0.6 (0.6–0.6)	2.0 (1.9–2.0)	1.0 (1.0–1.0)
Sodium, mg/100g	36.3 (35.7–36.9)	182.6 (179.5–185.7)	64.2 (63.4–65.0)	1118.0 (1082.4–1153.6)	381.4 (370.5–392.4)
% energy from protein	19.9 (19.8–20.0)	2.2 (2.1–2.2)	16.5 (16.4–16.6)	14.3 (14.2–14.4)	15.4 (15.4–15.5)
% energy from fat	14.9 (14.8–15.0)	44.1 (43.6–44.6)	20.5 (20.3–20.6)	26.2 (26.0–26.4)	21.6 (21.5–21.8)
% energy from saturate fat	6.3 (6.2–6.4)	14.2 (13.9–14.4)	7.8 (7.7–7.9)	9.6 (9.5–9.7)	8.1 (8.1–8.2)
% energy from carbohydrate	33.9 (33.8–34.1)	47.0 (46.5–47.5)	36.4 (36.3–36.6)	49.5 (49.3–49.7)	39.9 (39.7–40.0)
% energy from free sugars	2.4 (2.3–2.5)	35.5 (35.0–36.1)	8.7 (8.6–8.9)	18.6 (18.4–18.9)	12.0 (11.9–12.1)

^1^ *CI* Confidence intervals

### Socio-demographic characteristics of individuals with different relative intakes of processed foods

Table 4 shows mean percentage of energy from food processing groups across levels of socio-demographic variables. Results of multiple linear regression models exploring these associations, with simultaneous adjustment for gender, occupational social class, age and percentage of energy from alcohol are also shown. Women obtained significantly more energy than men from MPF, and MPF and PI combined; and significantly less energy form UPF. The only differences in intake by occupational social class was that those in the routine and manual class consumed significantly less energy from MPF, and MPF and PI than those in the managerial and professional class. There was a statistically significant positive association between age and percentage of energy from both MPF, and PI and MPF combined. There was a significant negative association between age and percentage of energy from UPF.

**Table 4:** Associations between socio-demographic variables and percentage of energy from processed-food groups; UK National Diet & Nutrition Survey, 2008-12

		% energy from unprocessed or minimally processed foods		% energy from processed ingredients		% energy from unprocessed or minimally processed foods AND processed ingredients		% energy from ultra-processed food products	
Variable	Level	% energy, mean (95 % CI)^1^	Adjusted^2^ coefficient (95% CI)	% energy, mean (95 % CI)	Adjusted coefficient (95 % CI)	% energy, mean (95 % CI)	Adjusted coefficient (95% CI)	% energy, mean (95 % CI)	Adjusted coefficient (95 % CI)
Gender	Male	26.2 (25.4 to 27.1)	Reference	13.2 (12.6 to 13.8)	Reference	39.4 (38.4 to 40.4)	Reference	53.3 (52.3 to 54.4)	Reference
	Female	29.3 (28.6 to 30.0)	2.28 (1.21 to 3.34)	13.5 (13.0 to 14.1)	−0.36 (−1.15 to 0.44)	42.9 (51.9 to 53.7)	1.92 (0.62 to 3.22)	52.8 (51.9 to 53.7)	−1.38 (−2.67 to −0.09)
Occupational social class	Managerial & prof.	28.4 (27.6 to 29.2)	Reference	12.9 (12.3 to 13.4)	Reference	41.3 (40.3 to 42.3)	Reference	52.0 (51.0 to 53.0)	Reference
	Intermediate	28.4 (27.3 to 29.4)	−0.83 (−2.10 to 0.44)	13.5 (12.8 to 14.2)	0.17 (−0.73 to 1.06)	41.9 (40.7 to 43.1)	−0.66 (−2.14 to 0.81)	53.3 (52.1 to 54.5)	0.34 (−1.12 to 1.79)
	Routine & manual	26.2 (25.2 to 27.3)	−2.62 (−3.88 to −1.35)	13.8 (12.9 to 14.7)	0.56 (−0.48 to 1.59)	40.0 (38.5 to 41.5)	−2.06 (−3.73 to −0.39)	54.7 (53.2 to 56.1)	1.60 (−0.05 to 3.26)
Age group^3^	18–29 years	23.5 (21.9 to 25.0)	0.16 (0.13 to 0.19)	13.2 (12.1 to 14.3)	0.02 (−0.01 to 0.04)	36.2 (34.3 to 38.0)	0.18 (0.15 to 0.22)	58.2 (56.3 to 60.2)	−0.18 (−0.21 to −0.14)
	30–39 years	25.7 (24.6 to 26.8)		13.2 (12.4 to 14.0)		38.9 (37.5 to 40.2)		55.9 (54.5 to 57.3)	
	40–49 years	27.1 (25.9 to 28.2)		13.4 (12.5 to 14.3)		40.4 (39.0 to 41.9)		52.2 (50.7 to 53.6)	
	50–59 years	29.7 (28.4 to 30.9)		13.4 (12.2 to 14.5)		43.0 (41.3 to 44.8)		49.7 (48.1 to 51.3)	
	60–69 years	32.5 (31.3 to 33.7)		12.7 (11.8 to 13.6)		45.2 (43.8 to 46.5)		49.0 (47.5 to 50.5)	
	70+ years	31.4 (30.1 to 32.7)		14.6 (13.7 to 15.5)		46.0 (44.5 to 47.5)		50.6 (49.0 to 52.2)	
All		27.8 (27.3 to 28.4)	NA	13.4 (13.0 to 13.8)	NA	41.2 (40.5 to 41.9)		53.1 (52.4 to 53.7)	NA

^1^ *CI* Confidence interval; ^2^linear regression coefficient simultaneously adjusted for gender, occupational social class, age and percentage of energy from alcohol; ^3^descriptive statistics are shown by age group, age was entered into linear regression models as a continuous variable

### Nutritional content of diets with different relative intakes of processed foods

Table 5 summarises the nutritional content of individual diets according to tertiles of percentage of energy from foods in the different food processing groups. For comparison, recommended population intake ranges suggested by the World Health Organization and Food and Agriculture Organization [24] in order to prevent diet-related chronic diseases are also shown. On only two occasions do 95 % confidence intervals indicate almost all members of a group achieve these recommendations. Those consuming the lowest intake of MPF achieve the recommended intake of protein and those with the highest intake of MPF and PI combined achieve the recommended intake of sodium.

**Table 5:** Intake of foods & nutrients according to tertiles of intake of food-processing groups; UK National Diet & Nutrition Survey, 2008−12

	Total daily, mean (95 % CI)^1^			Percent of energy derived from, mean (95 % CI)				
	Fruit & veg, portions	Fibre, g	Sodium, mg	Protein	Fat	Saturated fat	Carbohydrates	Free sugars
Tertiles of percentage of energy from unprocessed or minimally processed foods								
1 (lowest)	2.9 (2.8 to 3.1)	12.6 (12.1 to 13.0)	2541 (2454 to 2628)	14.6 (14.3 to 14.8)	33.5 (32.9 to 34.1)	12.6 (12.3 to 12.9)	46.0 (45.3 to 46.6)	13.6 (13.0 to 14.2)
2	4.0 (3.9 to 4.2)	14.3 (13.9 to 14.6)	2305 (2243 to 2368)	16.2 (15.9 to 16.4)	33.5 (33.0 to 34.0)	12.5 (12.3 to 12.8)	45.7 (45.1 to 46.3)	11.4 (10.9 to 11.8)
3 (highest)	4.9 (4.7 to 5.1)	14.4 (14.0 to 14.8)	1938 (1874 to 2001)	18.7 (18.3 to 19.1)	32.6 (32.0 to 33.1)	11.9 (11.6 to 12.2)	45.3 (44.6 to 45.9)	9.6 (9.2 to 10.1)
Adj^2^ coefficient (95 % CI)	0.07 (0.06 to 0.08)	0.07 (0.04 to 0.09)	−19.2 (−22.8 to −15.6)	0.18 (0.15 to 0.22)	−0.08 (−0.75 to 0.50)	−0.07 (−0.08 to −0.05)	−0.10 (−0.14 to −0.06)	−0.16 (−0.19 to −0.13)
Tertiles of percentage of energy from processed ingredients								
1 (lowest)	3.9 (3.7 to 4.1)	13.7 (13.3 to 14.1)	2315 (2243 to 2387)	16.8 (16.4 to 17.2)	32.2 (31.5 to 32.8)	11.7 (11.4 to 11.9)	44.7 (44.0 to 45.4)	11.6 (11.0 to 12.2)
2	4.1 (3.9 to 4.3)	14.0 (13.6 to 14.4)	2323 (2240 to 2406)	16.5 (16.2 to 16.8)	33.5 (33.0 to 34.0)	12.5 (12.2 to 12.8)	45.2 (44.6 to 45.7)	11.5 (11.1 to 12.0)
3 (highest)	3.9 (3.7 to 4.0)	13.6 (13.2 to 14.0)	2146 (2078 to 2214)	16.2 (15.9 to 16.5)	33.9 (33.4 to 34.5)	12.8 (12.5 to 13.1)	46.9 (46.3 to 47.5)	11.4 (10.9 to 11.9)
Adj coefficient (95 % CI)	0.00 (−0.01 to 0.01)	−0.02 (−0.06 to −1.89)	−9.8 (−15.4 to −4.3)	−0.07 (−0.10 to −0.04)	0.03 (−0.02 to 0.07)	0.02 (−0.01 to 0.04)	0.05 (−0.01 to 0.09)	−0.01 (−0.06 to 0.03)
Tertiles of percentage of energy from unprocessed or minimally processed foods AND processed ingredients								
1 (lowest)	3.1 (2.9 to 3.3)	12.9 (12.5 to 13.3)	2560 (2474 to 2646)	14.8 (14.5 to 15.1)	33.0 (32.4 to 33.6)	12.2 (11.9 to 12.5)	45.6 (44.9 to 46.3)	13.4 (12.8 to 14.0)
2	4.0 (3.8 to 4.2)	14.1 (13.7 to 14.5)	2323 (2258 to 2387)	16.5 (16.3 to 16.8)	33.5 (33.0 to 34.0)	12.7 (12.4 to 12.9)	45.4 (44.8 to 45.9)	11.0 (10.6 to 11.4)
3 (highest)	4.7 (4.5 to 4.9)	14.2 (13.8 to 14.7)	1907 (1848 to 1967)	18.1 (17.7 to 18.5)	33.1 (32.5 to 33.7)	12.2 (11.9 to 12.5)	45.9 (45.3 to 46.6)	10.2 (9.8 to 10.7)
Adj coefficient (95 % CI)	0.05 (0.04 to 0.06)	0.04 (0.02 to 0.06)	−17.1 (−20.4 to −13.8)	0.10 (0.07 to 0.13)	−0.05 (−0.08 to −0.02)	−0.04 (−0.05 to −0.03)	−0.05 (−0.09 to −0.02)	−0.11 (−0.14 to −0.09)
Tertiles of percentage of energy from ultra-processed food products								
1 (lowest)	4.6 (4.4 to 4.8)	13.9 (13.5 to 14.3)	1987 (1923 to 2051)	17.5 (17.1 to 17.9)	31.8 (31.2 to 32.5)	11.6 (11.3 to 11.9)	43.3 (42.6 to 44.0)	10.0 (9.6 to 10.4)
2	4.1 (3.9 to 4.2)	14.1 (13.7 to 14.5)	2288 (2223 to 2352)	16.5 (16.2 to 16.8)	33.3 (32.9 to 33.8)	12.6 (12.4 to 12.9)	45.9 (45.4 to 46.4)	11.5 (11.1 to 12.0)
3 (highest)	3.2 (3.0 to 3.3)	13.3 (12.9 to 13.8)	2512 (2425 to 2598)	15.5 (15.2 to 15.8)	34.5 (34.0 to 35.0)	12.8 (12.6 to 13.1)	47.8 (47.2 to 48.4)	13.1 (12.5 to 13.7)
Adj coefficient (95 % CI)	−0.05 (−0.06 to −0.04)	−0.04 (−0.06 to −0.02)	17.2 (13.9 to 20.6)	−0.10 (−0.13 to −0.07)	0.05 (0.02 to 0.08)	0.04 (0.03 to 0.05)	0.05 (0.01 to 0.08)	0.10 (0.08 to 0.13)
All								
Recommended^3^	At least 5.0	>25	<2000	10–15	15–30	<10	55–75	<10

^1^CI: confidence interval; ^2^linear regression coefficient adjusted for gender, occupational social class, age, and percentage energy from alcohol; ^3^population intake ranges recommended by the World Health Organization and Food & Agriculture Organization in order to prevent diet related chronic diseases

Table 5 also shows results of multiple linear regression models, adjusted for gender, occupational social class, age and percentage of energy from alcohol, of the associations between percentage of energy from food processing groups and all foods/nutrients studied. These are in expected directions with fruit and vegetable, fibre, and protein intake significantly decreasing, and sodium, fat, saturated fat, carbohydrate and sugar intake significantly increasing as UPF intake increased. The reverse associations were seen with MPF and PI intake combined. Associations between MFP alone and nutrients/foods followed the same pattern as MFP and PI combined, but the association for fat was not statistically significant. Fewer statistically significant associations were seen between PI intake and nutrient/food intake.

### Association between intake of processed foods and overweight and obesity

Results of linear regression models of the association between percentage of energy derived from food processing groups and BMI are shown in Table 6. Also shown are results of logistic regression models exploring change in odds of overweight and obesity combined, and obesity alone. These associations are adjusted for gender, occupational social class, age. Greater intake of PI was associated with lower BMI and reduced odds of overweight and obesity, and obesity. Greater intake of PI and MFP combined was associated with lower odds of overweight and obesity only. There were no significant associations between intake of MPF or UPF and markers of body weight.

**Table 6:** Associations between percentage of energy from processed-food groups and overweight and obesity; UK National Diet & Nutrition Survey, 2008–12

Percentage of energy from	Adjusted^1^ linear regression coefficient for body mass index (95% CI)^2^	Adjusted odds ratio of being overweight or obese (95% CI)	Adjusted odds ratio of being obese (95% CI)
Unprocessed or minimally processed foods	0.02 (−0.02 to 0.07)	1.00 (0.99 to 1.01)	1.00 (0.99 to 1.01)
Processed ingredients	−0.09 (−0.16 to -0.03)	0.97 (0.96 to 0.99)	0.98 (0.97 to 0.99)
Unprocessed or minimally processed foods AND processed ingredients	−0.02 (−0.06 to 0.02)	0.99 (0.98 to 0.99)	0.99 (0.98 to 1.00)
Ultra-processed food products	0.02 (−0.02 to 0.07)	1.01 (1.00 to 1.02)	1.01 (1.00 to 1.02)

^1^adjusted for gender, occupational social class, age and percentage of energy from alcohol; ^2^ *CI* Confidence intervals

## Discussion

### Summary of results

This is the first study we are aware of to explore correlates of processed food consumption, using individual-level data from a large, national cross-sectional sample. Unprocessed and minimally processed foods, and diets relatively high in these foods, tended to have the most healthful nutritional profile. Whilst UPF did not necessarily have the least healthful nutritional profile, diets relatively high in these foods did. Women and older people consumed a higher percentage of energy from MPF and a lower percentage of energy from UPF than men and younger people. Those living in lowest occupational social class households consumed a lower percentage of energy from MPF than those living the highest class households. Relative consumption of PI was inversely associated with all markers of body weight.

### Strengths and limitations of methods

Unlike previous work [7, 8, 11–13, 15–17], we used individual-level dietary data. Food diary data is likely to give a more accurate assessment of total dietary intake than previous methods [25, 26]. This should result in a reduction both in error and bias. Unlike household budget data, food diaries take food wastage into account, include unpackaged food and food eaten out of home, and do not assume all individuals within a household consume the same diet.

We used data from a large national cross-sectional survey, applying weighting to reduce any sampling and non-response bias. As such, our results are likely to be generalizable to the UK as a whole. They may also be applicable to similar international contexts.

Unlike many previous authors [6–8, 10–12, 14–17, 27], we have been explicit in how foods were coded to food processing groups. We found applying this coding harder than anticipated. More explicit information on the definitions of each group, or standard coding frameworks, may be useful. Our coding scheme (Additional file 1) could be a starting point for this.

We included alcoholic drinks in our calculations of total energy intake, because of the substantial contribution they can make to energy intake and adjusted for it in all models [19]. However, alcoholic drinks are specifically excluded from the food processing framework we used [7]. Further work is required to establish whether and how alcoholic drinks should be included in this framework.

Our data were cross-sectional and the only marker of health and disease on which we had information was BMI. This makes it difficult to draw firm conclusions on the impact of diets high or low in processed foods on health and disease. Future studies should make use of longitudinal data from cohorts with detailed information on morbidity and mortality.

### Comparison to previous findings and interpretation of findings

The overall balance of food intake according to degree of processing reported here is similar to other findings from developed countries. For example, 2012 US data found 61 % of energy was derived from UPF, 23 % from MPF and 16 % from PI [5]. In 2011 data from Canada, respective figures were 62 %, 26 % and 13 % [12] and in 2008 data from the UK, 63 %, 23 % and 14 % [11]. The differences from the current data (53 %, 28 % and 13 %) are likely mostly due to inclusion of energy from alcohol in our calculation of total energy intake.

As MPF have an energy density of around 10–20 % of other foods, it is not surprising that they make a much lower contribution to overall energy intake than other foods. Given current knowledge, it is difficult to confirm what a ‘safe’ or ‘balanced’ level of intake from each food processing group is. Future work should attempt to determine this.

Our finding that MPF have the most healthful nutritional profile reflect previous findings [5, 7, 8]. We did not confirm previous assertions that UPF have the least healthful profile [7, 8]. But we did confirm one previous finding [5] that PI have the least healthful profile.

Unlike previous work, we also studied the nutritional content of diets according to relative intake of all food processing groups and not just UPF [8]. Our results at the total diet level partly reinforced our findings at the food level, suggesting that diets relatively high in MPF (and in MPF and PI combined), tend to have the most healthful nutritional profile and diets high in UPF the least healthful profile. Public health messages aiming to maximise dietary quality could encourage both more consumption of MPF and less consumption of UPF.

Our finding that women and older people had higher relative intakes of MPF and UPF reflect the general patterns in dietary intake reported in NDNS – older adults and women tend to report more healthful diets [19]. An inverse association between socio-economic position and consumption of UPF was found in Brazil [7, 15], but not Canada [8]. We found that those living in households of the lowest occupational social class consumed less energy from MPF than others, but differences in UPF by social class were not found. Explanations for socio-economic differences in markers of healthy diet include differences in the relative affordability of ‘healthy’ versus ‘unhealthy’ products [11, 28]. Socio-economic differences in intake of fruit, vegetables, and some nutrients have been reported in NDNS [19]. Even if consumption of foods classified according to degree of food processing is confirmed as a predictor of health and disease, failure to consistently capture the extent of within-population differences in dietary intake may be a limitation of this approach.

We did not find a consistent association between intake of UPF and markers of body weight. It is not unusual to find no relationship between intake of energy-dense foods and body weight in cross-sectional studies (perhaps due to selective under-reporting and social desirability bias) [29]. However, previous studies have found a relationship between UPF intake and body weight [13]. Perhaps unexpectedly (given the nutritional profile of foods in this group), only higher intake of PI was consistently, inversely, associated with markers of overweight. Others have suggested that high intakes of PI may reflect home cooking [8] – which has been associated with better dietary quality and lower body weight [30–36]. It is possible that any benefits, in terms of nutritional content of diet, of high intake of MPF and low intake of UPF, do not translate to body weight.

### Implications of findings for research, policy and practice

Further work may be required to refine the framework we used to categorise foods, particularly to determine the role of alcohol in the framework. Given the difficulties we had coding foods, further guidance on exactly what foods fall into each group would be helpful. Standardised look-up files could be useful to facilitate future research.

Future work should focus on exploring longitudinal, as well as cross-sectional, associations between relative consumption of food processing groups and disease-related outcomes.

Further consideration of the limited socio-economic differences in intake of food processing groups may also be required. If these food groups do not consistently capture known socio-economic differences in diet, they may not be useful from a surveillance or public health point of view.

It has been suggested that the focus of current dietary guidelines on traditional food groups (e.g. fruits and vegetables; starchy foods and grains; dairy; non-dairy protein) [37] is inconsistent [7]. For example, both porridge made from oats and a commercial breakfast cereal would be categorised starchy foods and grains, but the nutritional content of these two foods could be very different. By focusing on the degree of food processing, rather than traditional food groups, it is suggested that associations with disease may become clearer [7]. The inconsistent associations between consumption of food processing groups and BMI found here do not provide strong support for this hypothesis.

It has been proposed that public health messages focusing on degree of processing, rather than traditional food groups, could be less confusing for consumers [7]. Whilst current guidance can certainly be confusing [38], this does not necessarily mean that focusing on food processing would be any less confusing – or more effective. Further work is required to determine whether degree of processing could contribute to useful public health messages for promotion of healthful diets.

## Conclusions

In a large national cross-sectional study of UK adults, MPF tended to have the most healthful nutritional profile. Diets relatively high in MPF and those relatively low in UPF had the most healthful nutritional profiles. These diets were more likely to be consumed by older people and those living in higher occupational social class households. Only higher intake of PI was consistently associated with markers of lower body weight.

## Additional file

Additional file 1:
**Coding of subsidiary food groups from National Diet and Nutrition Survey according to degree of industrial processing (Online supplemental material).** (DOCX 16 kb)

## Abbreviations

BMI: Body mass index
MPF: Unprocessed and minimally processed foods
NDNS: National diet & Nutrition Survey
UK: United Kingdom
PI: Processed ingredients
UPF: Ultra-processed food products
