# Supplementary material for: Characterisation of UK diets according to degree of food processing and associations with socio-demographics and obesity: cross-sectional analysis of UK National Diet and Nutrition Survey (2008–12)
Source: Int J Behav Nutr Phys Act. 2015 Dec 18;12:160. doi: 10.1186/s12966-015-0317-y (PMC4683717; doi:10.1186/s12966-015-0317-y)
Supplement: Additional file 1: — Coding of subsidiary food groups from National Diet and Nutrition Survey according to degree of industrial processing (Online supplemental material). (DOCX 16 kb) [file 12966_2015_317_MOESM1_ESM.docx]

**Additional file 1 – coding of subsidiary food groups from National Diet and Nutrition Survey according to degree of industrial processing (Online supplemental material)**

| **Subsidiary food group code** | **Subsidiary food group name** | **Food-processing group** |
| --- | --- | --- |
| 1C | Pizza | processed ingredient if just base; ultra-processed otherwise |
| 1D | Pasta (manufactured products and ready meals | ultra-processed |
| 1E | Pasta (other, including homemade dishes) | processed ingredient |
| 1F | Rice (manufactured products and ready meals) | ultra-processed |
| 1G | Rice (other, including homemade dishes) | processed ingredient |
| 1R | Other cereals | processed ingredient |
| 2R | White bread (not high fibre, not multiseed bread) | ultra-processed |
| 3R | Wholemeal bread | ultra-processed |
| 4R | Other bread | ultra-processed |
| 5R | High fibre breakfast cereals | ultra-processed |
| 6R | Other breakfast cereals (not high fibre) | ultra-processed |
| 7A | Biscuits(manufactured/retail) | ultra-processed |
| 7B | Biscuits (homemade) | processed ingredient |
| 8B | Fruit pies (manufactured) | ultra-processed |
| 8C | Fruit pies (homemade) | processed ingredient |
| 8D | Buns cakes and pastries (manufactured) | ultra-processed |
| 8E | Buns cakes and pastries (homemade) | processed ingredient |
| 9C | Cereal based milk puddings (manufactured) | ultra-processed |
| 9D | Cereal based milk puddings (homemade) | processed ingredient |
| 9E | Sponge puddings (manufactured) | ultra-processed |
| 9F | Sponge puddings (homemade) | processed ingredient |
| 9G | Other cereal based puddings (manufactured) | ultra-processed |
| 9H | Other cereal based puddings (homemade) | processed ingredient |
| 10R | Whole milk | minimally processed |
| 11R | Semi-skimmed milk | minimally processed |
| 12R | Skimmed milk | minimally processed |
| 13A | Infant formula | ultra-processed |
| 13B | Cream (including imitation cream) | ultra-processed if sweetened, imitation; processed ingredient otherwise |
| 13R | Other milk | ultra-processed if sweetened or fortified; processed ingredient if just dried; minimally processed otherwise |
| 14A | Cottage cheese | ultra-processed |
| 14B | Cheddar cheese | ultra-processed |
| 14R | Other cheese | ultra-processed |
| 15B | Yogurt | minimally processed if unsweetened, unfortified, no additions; ultra-processed otherwise |
| 15C | Fromage frais and other dairy desserts (manufactured) | ultra-processed |
| 15D | Dairy desserts (homemade) | processed ingredient |
| 16C | Manufactured egg products, including ready meals | ultra-processed |
| 16D | Other eggs and egg dishes, including homemade | minimally processed |
| 17R | Butter | processed ingredient |
| 18A | Polyunsaturated margarine | processed ingredient |
| 18B | Polyunsaturated oils | processed ingredient |
| 19A | Polyunsaturated low fat spread | processed ingredient |
| 19R | Low fat spread not polyunsaturated | processed ingredient |
| 20A | Block margarine | processed ingredient |
| 20B | Soft margarine not polyunsaturated | processed ingredient |
| 20C | Other cooking fats and oils not polyunsaturated | processed ingredient |
| 21A | Reduced fat spread (polyunsaturated) | processed ingredient |
| 21B | Reduced fat spread (not polyunsaturated) | processed ingredient |
| 22A | Ready meals/meal centres based on bacon and ham | ultra-processed |
| 22B | Other bacon and ham (including homemade dishes) | ultra-processed |
| 23A | Manufactured beef products (including ready meals) | ultra-processed |
| 23B | Other beef & veal (including homemade recipe dishes) | minimally processed |
| 24A | Manufactured lamb products (including ready meals) | ultra-processed |
| 24B | Other lamb (including homemade recipe dishes) | minimally processed |
| 25A | Manufactured pork products(including ready meals) | ultra-processed |
| 25B | Other pork (including homemade recipe dishes) | minimally processed |
| 26A | Manufactured coated chicken/turkey products | ultra-processed |
| 27A | Manufactured chicken products (including ready meals) | ultra-processed |
| 27B | Other chicken/turkey (including homemade recipe dishes) | minimally processed |
| 28R | Liver and dishes | ultra-processed if pate/processed; minimally processed otherwise |
| 29R | Burgers and kebabs purchased | ultra-processed |
| 30A | Ready meals based on sausages | ultra-processed |
| 30B | Other sausages (including homemade dishes) | ultra-processed |
| 31A | Meat pies and pastries (manufactured) | ultra-processed |
| 31B | Meat pies and pastries (homemade) | processed ingredient |
| 32A | Other meat products (manufactured including ready meals) | ultra-processed |
| 32B | Other meat (including homemade recipe dishes) | minimally processed |
| 33R | White fish coated or fried | minimally processed if any fish or fillet; ultra-processed if fish finger or fishcake |
| 34C | Manufactured white fish products (including ready meals) | ultra-processed |
| 34D | Other white fish (including homemade dishes) | ultra-processed if smoked or cured, minimally processed otherwise |
| 34E | Manufactured shellfish products (including ready meals) | ultra-processed |
| 34F | Other shellfish (including homemade dishes) | minimally processed |
| 34G | Manufactured canned tuna products (including ready meals) | ultra-processed |
| 34H | Other canned tuna(including homemade dishes) | ultra-processed |
| 35A | Manufactured oily fish products (including ready meals) | ultra-processed |
| 35B | Other oily fish (including homemade dishes) | minimally processed |
| 36A | Carrots (raw) | minimally processed |
| 36B | Salad and other raw vegetables | minimally processed |
| 36C | Tomatoes raw | minimally processed |
| 37A | Peas not raw | ultra-processed if canned with addition of salt/sugar; minimally processed otherwise |
| 37B | Green beans not raw | ultra-processed if canned with addition of salt/sugar; minimally processed otherwise |
| 37C | Baked beans | ultra-processed |
| 37D | Leafy green vegetables not raw | ultra-processed if canned with addition of salt/sugar; minimally processed otherwise |
| 37E | Carrots not raw | ultra-processed if canned with addition of salt/sugar; minimally processed otherwise |
| 37F | Tomatoes not raw | ultra-processed if preserved in oil; minimally processed otherwise |
| 37I | Beans and pulses (including ready meal & homemade dishes) | ultra-processed if canned with addition of salt/sugar or branded meal; processed ingredient if ingredient (flour); minimally processed otherwise |
| 37K | Meat alternatives (including ready meals and homemade dishes) | ultra-processed |
| 37L | Other manufactured vegetable products (including ready meals) | ultra-processed |
| 37M | Other vegetables (including homemade dishes) | minimally processed |
| 38A | Chips purchased including takeaway | ultra-processed |
| 38C | Other manufactured potato products fried/baked | ultra-processed |
| 38D | Other fried/roast potatoes(including homemade dishes) | minimally processed |
| 39A | Other potato products and dishes(manufactured) | ultra-processed |
| 39B | Other potatoes (including homemade dishes) | minimally processed |
| 40A | Apples and pears not canned | minimally processed |
| 40B | Citrus fruit not canned | minimally processed |
| 40C | Bananas | minimally processed |
| 40D | Canned fruit in juice | minimally processed |
| 40E | Canned fruit in syrup | ultra-processed |
| 40R | Other fruit not canned | ultra-processed if olives in brine or canned fruit filling; minimally processed otherwise |
| 41A | Sugar | processed ingredient |
| 41B | Preserves | ultra-processed |
| 41R | Sweet spreads fillings and icing | ultra-processed |
| 42R | Crisps and savoury snacks | ultra-processed |
| 43R | Sugar confectionery | ultra-processed |
| 44R | Chocolate confectionery | ultra-processed |
| 45R | Fruit juice | ultra-processed if sweetened or fortified; minimally processed otherwise |
| 47A | Liqueurs | not coded - alcohol |
| 47B | Spirits | not coded - alcohol |
| 48A | Wine | not coded - alcohol |
| 48B | Fortified wine | not coded - alcohol |
| 48C | Low alcohol and alcohol free wine | not coded - alcohol |
| 49A | Beers and lagers | not coded - alcohol |
| 49B | Low alcohol & alcohol free beer & lager | not coded - alcohol |
| 49C | Cider and perry | not coded - alcohol |
| 49D | Low alcohol & alcohol free cider & perry | not coded - alcohol |
| 49E | Alcoholic soft drinks (Alcopops) | not coded - alcohol |
| 50A | Beverages dry weight4 | ultra-processed |
| 50C | Soup (manufactured/retail) | ultra-processed |
| 50D | Soup (homemade) | minimally processed |
| 50E | Nutrition powders and drinks | ultra-processed |
| 50R | Savoury sauces pickles gravies & condiments | ultra-processed |
| 51A | Coffee (made up weight) | minimally processed |
| 51B | Tea (made up) | minimally processed |
| 51C | Herbal tea (made up) | minimally processed |
| 51D | Bottled water still or carbonated | minimally processed |
| 51R | Tap water only | minimally processed |
| 52A | Commercial toddlers drinks | ultra-processed |
| 52R | Commercial toddlers foods | ultra-processed |
| 53R | Ice cream | ultra-processed |
| 54A | Cod liver oil and other fish oils | processed ingredient |
| 54B | Evening primrose oil and other plant oils | processed ingredient |
| 54C | Single vitamins/minerals not Folic acid, iron, calcium | ultra-processed |
| 54D | Folic acid | ultra-processed |
| 54E | Iron only or with vitamin C | ultra-processed |
| 54F | Calcium only or with vitamin D | ultra-processed |
| 54G | Vitamins (two or more including multivitamins) no minerals | ultra-processed |
| 54H | Minerals (two or more including multiminerals) no vitamins | ultra-processed |
| 54I | Vitamins and minerals (including multivitamins & minerals) | ultra-processed |
| 54J | Non-nutrient supplements(including herbal) | ultra-processed |
| 54K | Other nutrient supplements | ultra-processed |
| 54L | Vitamin C | ultra-processed |
| 54M | Single vitamins/minerals not Folic acid, iron, calcium or vitamin C | ultra-processed |
| 54N | Cod liver oil and other fish oils (including with vitamins A, D, E) | ultra-processed |
| 54P | Multivitamins and/or minerals with omega ultra-processed | ultra-processed |
| 55R | Artificial sweeteners | processed ingredient |
| 56R | Nuts and seeds | ultra-processed if sweetened or salted; minimally processed otherwise |
| 57A | Soft drinks not low calorieconcentrated1 | ultra-processed |
| 57B | Soft drinks not low calorie carbonated | ultra-processed |
| 57C | Soft drinks not low calorie, ready to drink, still | ultra-processed |
| 58A | Soft drinks low calorieconcentrated1 | ultra-processed |
| 58B | Soft drinks low calorie carbonated | ultra-processed |
| 58C | Soft drinks low calorie, ready to drink, still | ultra-processed |
| 59R | Brown, granary and wheat germ bread | ultra-processed |
| 60R | 1% Milk | minimally processed |
| 61R | Smoothies | minimally processed |
